# Physical activity and its correlates among higher secondary school students in an urban district of Nepal

**DOI:** 10.1186/s12889-019-7230-2

**Published:** 2019-07-05

**Authors:** Kiran Thapa, Parash Mani Bhandari, Dipika Neupane, Shristi Bhochhibhoya, Janani Rajbhandari-Thapa, Ramjee Prasad Pathak

**Affiliations:** 1 Maharajgunj Medical Campus, Institute of Medicine, Tribhuvan University, Kathmandu, 44600 Nepal; 2 Department of Health and Exercise Sciences, University of Oklahoma, Norman, OK 73019 USA; 3 Department of Health Policy and Management, College of Public Health, University of Georgia, Athens, GA 30606 USA

**Keywords:** Physical activity, Adolescence, Correlates, Global physical activity questionnaire, Epidemiology, Nepal

## Abstract

**Background:**

Data on adolescents’ physical activity and determinants are scarce in Nepal. In this study, we aim to assess the level of physical activity, its correlates and the sedentary behavior of high school students in an urban district of Nepal.

**Methods:**

This is a cross-sectional study. Participants were selected using two-stage cluster sampling technique. We used Global Physical Activity Questionnaire (GPAQ) to collect information regarding physical activity and sedentary behavior. We also collected information about socio-demographic, academic, environmental and lifestyle-related factors. Data from 945 high school students from 23 randomly selected schools were analyzed. Logistic regression was used to identify correlates of low physical activity separately for male and female students.

**Results:**

Based on GPAQ classification, one out of five respondents reported low physical activity. The prevalence of low physical activity was 8% for males and 31% for females. About 31% of the adolescents and 14% of young adults did not meet the WHO recommendations of physical activity. Forty-seven percent of the total physical activity was borne by recreational activities. Correlates of low physical activity included school type and mode of transport among females, family support and drinking among males, and playground/park around home among both.

**Conclusions:**

The prevalence estimate of low physical activity among adolescents is high, with higher odds among females. Several different factors are associated with physical activity among males and females, therefore, interventions to promote physical activity in school may need to weigh these factors prior to/during implementation.

**Electronic supplementary material:**

The online version of this article (10.1186/s12889-019-7230-2) contains supplementary material, which is available to authorized users.

## Background

Evidence suggest that physical activity is required for healthful living because of its interrelationship with physical, mental and social well-being [1, 2]. Physical inactivity is an established modifiable risk factor of non-communicable diseases (NCDs) and is associated with an increase in all-cause mortality [3]. Marked changes in one’s physical, mental and behavioral functions including development of peer norms and social support during adolescence play important role in shaping activity preferences [4]. Literature has consistently shown that physical activity declines during adolescence [5]. More than four among five adolescents globally are insufficiently physically active [6].

Nepal, a low-income country located in South Asia, is in the phase of an epidemiological transition. Increasing urbanization and demographic transformation has led to an increase in lifestyle-related risk factors of chronic diseases such as low physical activity, sedentary behaviors, and sodium and fat consumption [7, 8]. World Health Organization (WHO) recommends at least 150 min of moderate-intensity physical activity or 75 min of vigorous-intensity physical activity daily for people aged 18–64 years and at least 60 min of moderate to vigorous physical activity for 5–17 years old [9]. But many Low- and Middle-Income Countries (LMICs) including Nepal do not have reliable and consistent data source to estimate regional disease burden related to physical inactivity which hinders evidence-based planning.

The STEPS Non-communicable Risk Factors survey - Nepal 2013 reports that 2.3% of the total study population aged 15–29 had low physical activity [8]. The proportion of females engaging in low levels of physical activity (0.7%) was notably lower than males (4.0%) [8]. In addition to this gender difference, physical activity and sedentary behavior among adolescents has been found to be associated with age, ethnicity, parental education, family income, parental and peer influence, self-efficacy, television watching, and availability of physical activity opportunities such as playground and walking trails [10–14]. These several socio-economic, psychological, and environmental factors vary across the regions of the world. Despite an array of published literatures on the benefits of physical activity, there has been minimal empirical research on physical activity and its determinants in LMICs including Nepal [15].

In this study, we aim to i) determine the prevalence of low physical activity (LPA) among high school students in an urban district of Nepal, and ii) assess correlates of physical activity.

## Methods

### Study design and setting

We conducted a quantitative cross-sectional study among high school students of randomly selected schools from Rupandehi district of Nepal. The study area is a south-western district of Nepal, which lies in Province No. 5 and has an area of 1360 km^2^. It is approximately 275 km south-west from Kathmandu, the capital city of Nepal. The district represents a typical urban setting in the context of LMICs - physical infrastructures and socio-demographics is changing rapidly due to urbanization and relocation of people from mid-hill mountains [16]. District Education Office (DEO) Report 2015 indicates that the district had a total of 92 higher secondary schools with 11,070 students in grade 11 and 12.

### Sample size and selection of participants

**Fig. 1:**
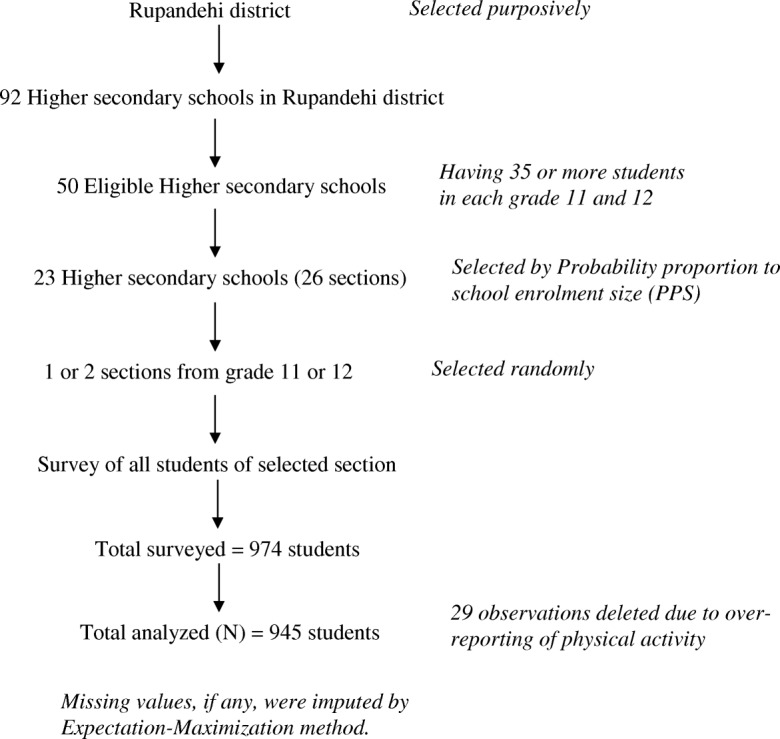
Sampling procedure

Considering the population size of 11,070, 95% confidence level, 3% margin of error and the population proportion of 0.5 (since the prevalence of physical inactivity among higher secondary students in Nepal is unknown), the optimal sample size was calculated at 974 [17]. We obtained a list of high schools and a number of students in each school from DEO. A high school having more than or equal to 35 students in each grade was considered eligible for data collection (sampling frame). We selected participants using a two-stage, cluster sample design to produce a representative sample of students. In the first sampling stage, among 50 eligible high schools, we selected 23 schools (primary sampling units) with probability proportional to size based on school enrolment. In the second sampling stage, we randomly selected entire classes by lottery method. All the students present on the day of data collection were asked to participate and none of them refused to take part in the research. The figure showing sampling procedure is shown in Fig. 1. The data were collected through the month of August of 2015. No efforts were made to contact students absent on the day of data collection.

### Measures/outcomes

We used the local (Nepali) version of Global Physical Activity Questionnaire (GPAQ) version 2 to collect information about physical activity and sedentary behavior [18, 19]. GPAQ v2, which has been shown to have low-to-moderate validity and generally acceptable reliability in Bangladesh and Vietnam [20, 21], is a suitable tool to assess physical activity in developing countries [22]. GPAQ collects information about physical activity in three domains, namely, work (paid or unpaid work outside of home), travel to and from places, and recreation or leisure time. Questions about work and recreational physical activity included vigorous and moderate level activities. We used physical activity show cards (pictures of different activities and sports) developed by WHO after contextual modification to ensure that activities were rightly classified as moderate and vigorous. Based on GPAQ analysis guide, we converted the responses to Metabolic Equivalent to Task (MET)-minutes/week [23]. The Total Physical Activity (TPA) score was calculated by adding the score of work MET-minutes/week, travel MET-minutes/week and recreational MET-minutes/week. Consequently, physical activity level was classified as low, moderate and high based on different combination criteria [24]. Furthermore, we also identified participants who met the minimum WHO recommendation for physical activity [9]. For this, we classified respondents aged less than 18 years as ‘adolescents’ and 18 or over as ‘young adults’. For adolescents, based on the WHO recommendation for physical activity for 5–17 years old, the cut-off value of 1680 MET-minutes/week was set and used (calculation shown in Table 1 footnote) [9]. And for young adults, the standard WHO cut-off of 600 MET-minutes/week was used. Sedentary behavior was assessed using the measure of sitting time per day, however, this excludes time spent in school (which is approximately 6 h a day) and sleeping.

**Table 1:** Population meeting WHO recommendation for physical activity stratified by age and sex [Data shown as percentage (95% confidence interval)]

	N	Population meeting WHO recommendation for physical activity (%)			
		Adolescents (< 18 years) (*n* = 638)		Young adults (≥18 years) (*n* = 307)	
		Male (*n* = 280)	Female (*n* = 358)	Male (*n* = 471)	Female (*n* = 474)
All	945	85.4 (81.3–89.5)	56.4 (51.3–61.5)	96.9 (94.4–99.4)	72.4 (64.3–80.5)
Socio-demographic variables					
Ethnicity					
Brahmin/Chhetri	575	83.8 (78.7–88.9)	56.8 (50.4–63.2)	95.2 (91.1–99.3)	80.9 (69.7–92.1)
Aadibasi/Janajati	245	94.1 (87.6–100.0)	51.5 (41.8–61.3)	100.0 (100.0–100.0)	66.7 (53.4–80.0)
Others	125	81.3 (67.8–94.8)	70.0 (53.6–86.4)	97.6 (93.0–100.0)	66.7 (46.5–86.9)
Family type					
Nuclear	682	85.9 (81.2–90.7)	56.8 (50.7–62.9)	95.8 (92.5–99.1)	77.3 (67.8–86.8)
Non-nuclear	263	83.8 (75.4–92.2)	55.4 (45.7–65.1)	100.0 (100.0–100.0)	63.4 (48.7–78.1)
Educational status of father					
Illiterate	40	100.0 (100.0–100.0)	71.4 (37.9–100.0)	91.7 (76.1–100.0)	70.0 (41.6–98.4)
Primary	122	89.2 (79.2–99.2)	65.4 (47.1–83.7)	97.1 (91.5–100.0)	76.0 (59.3–92.7)
Secondary	398	88.9 (83.4–94.4)	57.4 (49.6–65.2)	96.0 (91.6–100.0)	69.0 (55.0–83.0)
High school and above	385	78.3 (70.5–86.2)	53.5 (46.0–61.0)	98.6 (95.9–100.0)	74.4 (60.7–88.1)
Educational status of mother					
Illiterate	141	92.1 (83.5–100.0)	68.8 (52.8–84.9)	95.7 (89.9–100.0)	66.7 (47.8–85.6)
Primary	184	83.9 (74.8–93.1)	63.3 (49.8–76.8)	100.0 (100.0–100.0)	75.0 (59.0–91.0)
Secondary	408	86.9 (80.9–92.9)	54.7 (47.2–62.2)	93.9 (88.1–99.7)	70.0 (57.3–82.7)
High school and above	212	79.3 (68.9–89.7)	52.3 (42.8–61.8)	100.0 (100.0–100.0)	85.7 (67.4–100.0)
Academic variables					
Type of school					
Public	330	92.3 (86.8–97.8)	66.1 (57.2–75.0)	100.0 (100.0–100.0)	75.4 (64.6–86.2)
Private	615	82.0 (76.5–87.5)	52.2 (46.0–58.4)	95.1 (91.3–98.9)	69.1 (56.9–81.3)
Grade of study					
11	331	88.6 (83.2–94.0)	51.0 (43.0–59.0)	97.2 (91.8–100.0)	64.3 (39.2–89.4)
12	614	82.4 (76.3–88.5)	60.3 (53.7–66.9)	96.8 (94.0–99.6)	73.5 (64.9–82.1)
Subject of study					
Education	171	96.4 (89.5–100.0)	65.2 (53.7–76.7)	100.0 (100.0–100.0)	72.5 (58.7–86.3)
Humanities	24	100.0 (100.0–100.0)	50.0 (1.0–99.0)	100.0 (100.0–100.0)	81.8 (59.0–100.0)
Management	298	91.2 (86.0–96.4)	52.8 (44.0–61.6)	100.0 (100.0–100.0)	54.5 (33.7–75.3)
Science	452	77.8 (70.8–84.8)	55.8 (48.2–63.4)	94.5 (90.2–98.8)	79.1 (67.0–91.3)
Time of study					
Morning	372	93.1 (88.2–98.0)	64.6 (56.4–72.8)	100.0 (100.0–100.0)	69.4 (58.8–80.0)
Day	573	81.0 (75.3–86.8)	51.8 (45.3–58.3)	95.1 (91.3–98.9)	77.3 (64.9–89.7)
Environmental variables					
Mode of transport to school					
Walking	339	90.6 (85.0–96.2)	58.6 (49.9–67.3)	95.6 (90.7–100.0)	57.1 (42.1–72.1)
Cycle	134	89.6 (81.0–98.2)	85.3 (73.4–97.2)	100.0 (100.0–100.0)	94.4 (83.8–100.0)
Motorcycle/Four-wheeled	472	79.4 (72.3–86.5)	50.2 (43.3–57.1)	96.6 (92.8–100.0)	76.8 (65.7–87.9)
Extracurricular activities at school					
Yes	654	85.9 (80.7–91.1)	56.5 (50.6–62.4)	97.6 (94.9–100.0)	73.9 (64.9–82.9)
No	291	84.5 (77.7–91.3)	56.2 (45.9–66.5)	95.6 (90.7–100.0)	66.7 (47.8–85.6)
Playground at school					
Yes	793	85.2 (80.4–90.0)	57.4 (52.1–62.7)	97.1 (94.3–99.9)	71.9 (63.7–80.1)
No	152	85.9 (77.8–94.0)	44.4 (25.7–63.1)	96.2 (91.0–100.0)	100.0 (100.0–100.0)
Playground or park around home					
Yes	645	86.6 (81.8–91.4)	57.6 (51.4–63.8)	97.7 (95.1–100.0)	74.0 (64.2–83.8)
No	300	82.6 (74.6–90.6)	53.9 (44.8–63.0)	95.0 (89.5–100.0)	69.2 (54.7–83.7)
Adequate space to play or walk around home					
Yes	709	86.9 (82.4–91.4)	59.0 (53.1–64.9)	97.9 (95.5–100.0)	70.5 (61.0–80.0)
No	236	80.6 (71.1–90.1)	48.9 (38.6–59.2)	94.1 (87.6–100.0)	78.6 (63.4–93.8)
Family support to physical activity					
Yes	890	86.7 (82.6–90.8)	56.5 (51.2–61.8)	96.7 (94.1–99.3)	71.2 (62.8–79.6)
No	55	64.7 (42.0–87.4)	56.0 (36.6–75.5)	100.0 (100.0–100.0)	100.0 (100.0–100.0)
Peer support to physical activity					
Yes	900	86.6 (82.5–90.7)	56.8 (51.5–62.1)	96.8 (94.3–99.3)	71.9 (63.7–80.1)
No	45	66.7 (44.9–88.5)	50.0 (29.1–70.9)	100.0 (100.0–100.0)	100.0 (100.0–100.0)
Lifestyle-related variables					
Current smoker					
Yes	30	100.0 (100.0–100.0)	0.0 (0.0–0.0)	95.5 (86.8–100.0)	100.0 (100.0–100.0)
No	915	85.0 (80.8–89.2)	56.4 (51.3–61.5)	97.0 (94.4–99.6)	72.2 (64.0–80.4)
Current drinker					
Yes	42	80.0 (59.8–100.0)	100.0 (100.0–100.0)	100.0 (100.0–100.0)	0.0 (0.0–0.0)
No	903	85.7 (81.5–89.9)	56.3 (51.2–61.5)	96.4 (93.6–99.2)	72.4 (64.3–80.5)
Screen time					
Moderate	599	89.7 (85.2–94.2)	55.2 (48.7–61.7)	97.4 (94.5–100.0)	73.3 (62.1–84.5)
Excessive	346	78.1 (70.2–86.0)	58.5 (50.2–66.8)	96.0 (91.6–100.0)	71.4 (59.6–83.2)

Note: For adolescents aged less than 17 years, the cut-off value of 1680 MET-minutes/week was used. The World Health Organization (*WHO*) recommends at least 60 min of moderate to vigorous physical activity daily for adolescents aged 5–17 years old. Assuming the intensity of activity to be 4, the total MET-minutes per week is 60*4*7 = 1680 MET-minutes/week. For those aged 18–64 years old, the WHO recommendation of 600 MET-minutes/week is used

### Study variables

We collected information on four major groups of variables: socio-demographic, academic, environmental and lifestyle related variables. Socio-demographic variables included age, sex, ethnicity, type of family, and educational status of parents; academic variables included type of school, grade of study, subject of study (stream), and time of study; environmental variables included mode of transport to school, extracurricular activities at school, playground at school, playground or park around home, adequate space to play or walk around home, family support to physical activity, and peer support to physical activity; and lifestyle-related variables included smoking habit, drinking habit, and screen time. We divided ethnic groups into three categories viz. Brahmin/Chhetri, Aadibasi/Janajati, and “Others” based on the Nepal Demographic and Health Survey (NDHS) [7]. “Others” included ethnic minorities such as Dalit, Madhesi, Dashnami, Muslim, and Sanyasi. We classified educational status based on the Central Bureau of Statistics report, Nepal [25]. We divided school type into ‘public’ and ‘private’ based on the predominant school systems in Nepal. In Nepal, after students complete Secondary Education Examination (SEE), they have to the choose the stream (subject) so as to enrol into a two-year high school course usually offered during morning and/or during the day [26]. We categorized academic variables based on these local contexts. We adopted environmental variables from a similar study conducted in Nepal [10]. The environmental variables assess the physical activity opportunities available in school and around home. We assessed smoking and alcohol consumption based on the Yes/No responses to ‘Do you smoke?’ and ‘Do you drink?’ respectively. To assess screen time, participants were asked three questions about how much time they spend (‘< 2 h’ and ‘≥ 2 hours’) in a typical day watching television, playing video-game, and using a computer. Those who reported as spending less than 2 h in each audio-visual device were categorized as having ‘moderate screen time’, and those who reported as spending 2 h or more in any of the audio-visual device were categorized as having ‘excessive screen time’.

### Statistical analysis

In order to minimize errors, we arranged, coded and cleaned each questionnaire before entering into Epi Data 3.1 which was then exported to SPSS version 20 for further analysis. We followed GPAQ analysis guide to clean and analyze the data [23]. Though we collected data from 974 participants, we carried out the statistical analysis among 945 participants. We removed 29 questionnaires during data cleaning because participants over-reported the amount of physical activity (exceeded the maximum possible value i.e. 24 h/day). We replaced the missing fields, if any, with Expectation Maximization (E-M) method [27]. The E-M algorithm uses current estimate of the parameter to find expected data (E-step) followed by maximization of the likelihood estimate of the obtained parameter (M-step). We stratified data according to sex so that we could identify gender differences in physical activity and the associated factors. We reported categorical variables as percentages (95% confidence interval) and continuous variables as mean ± standard deviation or median (25th percentile, 75th percentile). Unadjusted and adjusted odds ratios (ORs) were calculated at 95% CI for LPA compared to moderate to vigorous physical activity (MVPA), a single measure obtained by combining the moderate and high physical activity level. A *p* < 0.05 was considered to be statistically significant.

### Ethical approval

We obtained technical and ethical approval from Department of Community Medicine and Public Health, Maharajgunj Medical Campus and Nepal Health Research Council (Ref. No. 158, 2015). The requirement of parental consent was waived given the non-interventional and non-invasive nature of the study. We briefed school principals about the objective of the study and received permission from them through phone calls and face-to-face meetings to conduct the study. We shared objectives of the study among the participants and took written informed consent from each of them prior to data collection. Privacy and confidentiality of the information was ensured throughout the research process. The recorded data were only used for the purpose of this research.

## Results

### Socio-demographic, academic, environmental and lifestyle-related information

Table 1 shows the characteristics of the study population. Respondents were almost equally split between males and females. The age ranged from 15 to 21 years with the mean of 17.16 ± 1.01 years and majority (65%) in the age group of 15–17 years. Most of the participants were Brahmin/Chhetri (61%), lived in nuclear family (72%), and had parents who completed secondary level of education (42% fathers and 43% mothers). There were high proportion of respondents from private schools (65%), grade 12 (65%), science stream (48%), and who studied during daytime (61%). About half of the respondents used motorcycle or four-wheeled vehicle on their commute to school. Majority of the participants reported of having extracurricular activities at school (69%), playground at school (84%), playground or park around home (68%), and adequate space to play or walk around home (75%). Approximately 94 and 95% of the respondents reported of having family support and peer support to physical activity respectively. Around 3% consumed tobacco and 4% were alcohol users. About 37% reported excessive screen viewing.

### Burden of physical inactivity

While about 97% of the young adult males (age ≥ 18 years) met the minimum WHO recommendation for physical activity (≥600 MET-minutes/week), only about 72% of the young adult females met the criteria. Similarly, about 85% of the adolescent males (age <18 years) and 56% of the adolescent females (age <18 years) met the criteria we set based on WHO recommendation for physical activity for 5–17 years old (Table 1). According to GPAQ classification, we found that almost one-fifth of the participants reported low physical activity (LPA). Among males, the figure was around 8%, while in females it was 31% (Table 2). Logistic regression revealed that females were five times more likely (OR: 5.12, 95% CI: 3.49, 7.52) to report LPA than males. Similarly, 27 and 54% of the respondents were found to be engaged in moderate and high level of physical activity. The median sitting time per day was 240 min while the mean sitting time was 282.93 ± 206.90 min per day (Table 3). There was no significant difference between the sitting time of males (280.04 ± 209.56 min/day) and females (285.81 ± 204.40 min/day), t(943) = 0.43, *p* = 0.78.

**Table 2:** GPAQ classification of physical activity stratified by sex [Data shown as percentage (95% confidence interval)]

	N	GPAQ classification					
		Low physical activity (%)		Moderate physical activity (%)		High physical activity (%)	
		Male (*n* = 471)	Female (*n* = 474)	Male (*n* = 471)	Female (*n* = 474)	Male (*n* = 471)	Female (*n* = 474)
All	945	8.1 (5.6–10.5)	31.0 (26.8–35.2)	19.3 (15.8–22.9)	34.2 (29.9–38.4)	72.6 (68.6–76.6)	34.8 (30.5–39.1)
Socio-demographic variables							
Age							
15–17 years	616	8.9 (5.5–12.3)	31.4 (26.5–36.3)	20.8 (16.0–25.7)	34.0 (29.0–39.0)	70.3 (64.8–75.7)	34.6 (29.6–39.6)
17–19 years	304	6.8 (3.2–10.4)	31.9 (23.3–40.4)	17.8 (12.4–23.2)	35.4 (26.6–44.2)	75.4 (69.3–81.5)	32.7 (24.1–41.4)
19–21 years	25	9.1 (0.0–26.1)	14.3 (0.0–32.6)	9.1 (0.0–26.1)	28.6 (4.9–52.2)	81.8 (59.0–100.0)	57.1 (31.2–83.1)
Ethnicity							
Brahmin/Chhetri	575	7.3 (4.4–10.2)	28.1 (22.8–33.4)	21.3 (16.6–25.9)	37.2 (31.5–43.0)	71.4 (66.3–76.5)	34.7 (29.0–40.3)
Aadibasi/Janajati	245	6.3 (1.4–11.1)	37.6 (29.8–45.4)	14.6 (7.5–21.6)	31.5 (24.1–39.0)	79.2 (71.0–87.3)	30.9 (23.5–38.3)
Others	125	13.5 (5.7–21.3)	27.5 (15.2–39.7)	17.6 (8.9–26.2)	25.5 (13.5–37.5)	68.9 (58.4–79.5)	47.1 (33.4–60.8)
Family type							
Nuclear	682	8.0 (5.2–10.8)	29.2 (24.3–34.1)	20.0 (15.8–24.2)	33.4 (28.4–38.5)	72.0 (67.3–76.7)	37.3 (32.1–42.6)
Non-nuclear	263	8.3 (3.4–13.2)	35.2 (27.4–43.1)	17.4 (10.6–24.1)	35.9 (28.0–43.8)	74.4 (66.6–82.2)	28.9 (21.4–36.3)
Educational status of father							
Illiterate	40	4.3 (0.0–12.7)	29.4 (7.8–51.1)	13.0 (0.0–26.8)	11.8 (0.0–27.1)	82.6 (67.1–98.1)	58.8 (35.4–82.2)
Primary	122	7.0 (1.1–13.0)	29.4 (16.9–41.9)	15.5 (7.1–23.9)	29.4 (16.9–41.9)	77.5 (67.7–87.2)	41.2 (27.7–54.7)
Secondary	398	8.0 (4.2–11.7)	31.0 (24.5–37.4)	17.4 (12.2–22.7)	33.5 (26.9–40.1)	74.6 (68.6–80.6)	35.5 (28.8–42.2)
High school and above	385	9.1 (4.8–13.3)	31.6 (25.3–37.9)	23.9 (17.6–30.2)	37.8 (31.2–44.4)	67.0 (60.1–74.0)	30.6 (24.4–36.9)
Educational status of mother							
Illiterate	141	7.1 (1.6–12.5)	28.6 (16.7–40.4)	17.6 (9.5–25.8)	25.0 (13.7–36.3)	75.3 (66.1–84.5)	46.4 (33.4–59.5)
Primary	184	7.4 (2.5–12.5)	31.2 (20.8–41.5)	16.8 (9.7–23.9)	31.2 (20.8–41.5)	75.7 (67.6–83.8)	37.7 (26.8–48.5)
Secondary	408	8.5 (4.5–12.5)	32.7 (26.5–38.9)	21.8 (15.9–27.7)	35.0 (28.7–41.3)	69.7 (63.1–76.3)	32.3 (26.1–38.5)
High school and above	212	8.8 (3.0–14.6)	28.9 (20.8–37.0)	18.7 (10.7–26.7)	38.8 (30.2–47.5)	72.6 (63.4–81.7)	32.2 (23.9–40.6)
Academic variables							
Type of school							
Public	330	4.4 (1.2–7.5)	24.1 (17.7–30.5)	11.9 (6.9–16.9)	26.5 (19.8–33.1)	83.8 (78.0–89.5)	49.4 (41.9–56.9)
Private	615	10.0 (6.6–13.3)	34.9 (29.5–40.2)	23.2 (18.5–27.8)	38.5 (33.0–44.0)	66.9 (61.7–72.1)	26.6 (21.7–31.6)
Grade of study							
11	331	7.7 (3.7–11.8)	37.4 (30.0–44.9)	20.2 (14.2–26.3)	33.1 (25.9–40.4)	72.0 (65.2–78.8)	29.4 (22.5–36.4)
12	614	8.3 (5.2–11.3)	27.7 (22.7–32.6)	18.8 (14.4–23.2)	34.7 (29.4–40.0)	72.9 (67.9–77.9)	37.6 (32.2–43.0)
Subject of study							
Education	171	1.5 (0.0–4.5)	28.3 (19.7–36.9)	21.5 (11.5–31.5)	27.4 (18.9–35.8)	76.9 (66.7–87.2)	44.3 (34.9–53.8)
Humanities	24	0.0 (0.0–0.0)	26.7 (4.3–49.0)	0.0 (0.0–0.0)	20.0 (0.0–40.2)	100.0 (100.0–100.0)	53.3 (28.1–78.6)
Management	298	6.5 (2.6–10.5)	41.4 (33.4–49.4)	16.3 (10.5–22.2)	25.5 (18.4–32.6)	77.1 (70.5–83.8)	33.1 (25.4–40.8)
Science	452	11.1 (7.1–15.0)	25.5 (19.6–31.4)	21.3 (16.2–26.4)	44.7 (38.0–51.5)	67.6 (61.8–73.5)	29.8 (23.6–36.0)
Time of study							
Morning	372	5.3 (1.9–8.7)	32.7 (26.2–39.1)	11.8 (6.9–16.6)	21.8 (16.1–27.5)	82.9 (77.3–88.6)	45.5 (38.7–52.4)
Day	573	9.6 (6.3–13.0)	29.8 (24.3–35.2)	23.6 (18.8–28.4)	43.4 (37.5–49.3)	66.8 (61.5–72.1)	26.8 (21.6–32.1)
Environmental variables							
Mode of transport to school							
Walking	339	6.9 (3.1–10.7)	33.3(26.1–40.5)	20.1 (14.2–26.1)	32.7 (25.6–39.9)	73.0 (66.4–79.6)	33.9 (26.7–41.2)
Cycle	134	3.7 (0.0–7.7)	9.6 (1.6–17.6)	11.0 (4.2–17.7)	26.9 (14.9–39.0)	85.4 (77.7–93.0)	63.5 (50.4–76.5)
Motorcycle/Four-wheeled	472	10.7 (6.6–14.8)	33.9 (28.1–39.6)	21.9 (16.3–27.4)	36.6 (30.7–42.5)	67.4 (61.2–73.7)	29.6 (24.0–35.2)
Extracurricular activities at school							
Yes	654	7.5 (4.5–10.5)	31.6 (26.8–36.4)	21.8 (17.1–26.6)	33.2 (28.4–38.1)	70.6 (65.4–75.9)	35.2 (30.3–40.1)
No	291	9.0 (4.8–13.2)	29.2 (20.8–37.6)	15.2 (9.9–20.4)	37.2 (28.3–46.1)	75.8 (69.6–82.1)	33.6 (24.9–42.3)
Playground at school							
Yes	793	8.0 (5.2–10.9)	31.0 (26.7–35.3)	21.6 (17.2–25.9)	33.9 (29.5–38.3)	70.4 (65.6–75.2)	35.1 (30.6–39.5)
No	152	8.1 (3.3–13.0)	31.0 (14.2–47.9)	13.0 (7.1–19.0)	37.9 (20.3–55.6)	78.9 (71.6–86.1)	31.0 (14.2–47.9)
Playground or park around home							
Yes	645	5.8 (3.3–8.4)	28.4 (23.5–33.4)	20.6 (16.2–25.0)	33.4 (28.3–38.6)	73.5 (68.7–78.3)	38.1 (32.8–43.4)
No	300	13.0 (7.6–18.5)	36.4 (28.8–44.0)	16.4 (10.4–22.5)	35.7 (28.1–43.3)	70.5 (63.2–77.9)	27.9 (20.8–35.0)
Adequate space to play or walk around home							
Yes	709	7.1 (4.4–9.8)	30.1 (25.3–34.8)	18.4 (14.4–22.5)	31.7 (26.9–36.6)	74.5 (70.0–79.1)	38.2 (33.2–43.2)
No	236	11.0 (5.4–16.7)	33.9 (25.4–42.4)	22.0 (14.6–29.5)	41.5 (32.6–50.4)	66.9 (58.5–75.4)	24.6 (16.8–32.3)
Family support to physical activity							
Yes	890	7.2 (4.8–9.6)	30.9 (26.6–35.2)	19.3 (15.6–22.9)	34.2 (29.8–38.6)	73.5 (69.4–77.6)	34.9 (30.5–39.3)
No	55	24.0 (7.3–40.7)	33.3 (16.5–50.2)	20.0 (4.3–35.7)	33.3 (16.5–50.2)	56.0 (36.5–75.5)	33.3 (16.5–50.2)
Peer support to physical activity							
Yes	900	7.3 (4.9–9.7)	31.1 (26.8–35.4)	19.1 (15.5–22.7)	33.8 (29.4–38.1)	73.6 (69.5–77.6)	35.1 (30.7–39.5)
No	45	23.8 (5.6–42.0)	29.2 (11.0–47.4)	23.8 (5.6–42.0)	41.7 (21.9–61.4)	52.4 (31.0–73.7)	29.2 (11.0–47.4)
Lifestyle-related variables							
Current smoker							
Yes	30	10.3 (0.0–21.4)	0.0 (0.0–0.0)	13.8 (1.2–26.3)	100.0 (100.0–100.0)	75.9 (60.3–91.4)	0.0 (0.0–0.0)
No	915	7.9 (5.4–10.4)	31.1 (26.9–35.2)	19.7 (16.0–23.4)	34.0 (29.8–38.3)	72.4 (68.2–76.6)	34.9 (30.6–39.2)
Current drinker							
Yes	42	17.1 (5.6–28.6)	0.0 (0.0–0.0)	9.8 (0.7–18.8)	100.0 (100.0–100.0)	73.2 (59.6–86.7)	0.0 (0.0–0.0)
No	903	7.2 (4.8–9.7)	31.1 (26.9–35.2)	20.2 (16.4–24.0)	34.0 (29.8–38.3)	72.6 (68.3–76.8)	34.9 (30.6–39.2)
Screen time							
Moderate	599	8.2 (5.0–11.3)	28.2 (23.1–33.2)	19.0 (14.6–23.5)	38.4 (32.9–43.8)	72.8 (67.7–77.9)	33.4 (28.1–38.7)
Excessive	346	7.9 (3.9–11.9)	36.1 (28.9–43.3)	19.8 (13.9–25.6)	26.6 (20.0–33.3)	72.3 (65.7–78.9)	37.3 (30.0–44.6)

**Table 3:** Domain specific physical activity scores and sitting time [Data shown as median (25th percentile, 75th percentile)]

	N	Physical activity (Median MET-minutes per week)				Median sitting time (minutes per day)
		Work related (*n* = 155)^a^	Travel related (*n* = 945)	Recreation related (*n* = 945)	Total	
All	945	0 (0, 0)	1120 (240, 2160)	1680 (180, 3960)	3480 (1320, 6960)	240 (120, 388)
Socio-demographic variables						
Sex						
Male	471	0 (0, 0)	1680 (560, 2520)	2880 (1260, 5640)	5360 (3080, 9240)	240 (120, 375)
Female	474	0 (0, 0)	720 (0, 1680)	600 (0, 2180)	2000 (600, 4330)	240 (120, 390)
Age						
15–17 years	616	0 (0, 0)	960 (240, 2100)	1680 (130, 3480)	3210 (1090, 6478)	240 (120, 393)
17–19 years	304	0 (0, 0)	1440 (259, 2520)	1680 (262, 4755)	3880 (1835, 8400)	240 (120, 360)
19–21 years	25	0 (0, 0)	1440 (0, 2520)	1920 (810, 4830)	5580 (1980, 8580)	180 (90, 282)
Ethnicity						
Brahmin/Chhetri	575	0 (0, 0)	1200 (360, 2160)	1680 (240, 3680)	3420 (1440, 6600)	247 (120, 420)
Aadibasi/Janajati	245	0 (0, 0)	840 (0, 2090)	1440 (0, 3840)	3120 (890, 7030)	195 (120, 344)
Others	125	0 (0, 0)	1600 (0, 2520)	2320 (520, 5280)	5040 (2340, 9660)	240 (120, 378)
Family type						
Nuclear	682	0 (0, 0)	1200 (240, 2240)	1780 (230, 4320)	3660 (1400, 7200)	240 (120, 370)
Non-nuclear	263	0 (0, 0)	900 (160, 2160)	1440 (160, 3640)	3240 (1276, 6680)	270 (120, 405)
Educational status of father						
Illiterate	40	0 (0, 0)	1740 (520, 3630)	2760 (375, 4845)	7260 (1737, 10,950)	202 (83, 300)
Primary	122	0 (0, 0)	1440 (440, 2930)	2520 (328, 5310)	5760 (2010, 8890)	200 (120, 315)
Secondary	398	0 (0, 0)	1160 (240, 2520)	1680 (175, 4110)	3600 (1380, 6990)	240 (120, 370)
High school and above	385	0 (0, 0)	840 (160, 1800)	1440 (30, 3360)	2840 (1100, 5520)	270 (120, 450)
Educational status of mother						
Illiterate	141	0 (0, 0)	1440 (280, 2520)	2040 (360, 4320)	4560 (2000, 8880)	190 (120, 330)
Primary	184	0 (0, 0)	1440 (210, 2520)	2160 (315, 4725)	4548 (1760, 8595)	210 (120, 330)
Secondary	408	0 (0, 0)	988 (240, 2145)	1440 (80, 3575)	3120 (1080, 6285)	243 (120, 393)
High school and above	212	0 (0, 0)	1120 (240, 2085)	1490 (65, 3670)	3070 (1090, 5910)	275 (120, 480)
Academic variables						
Type of school						
Public	330	0 (0, 0)	1440 (280, 2880)	2400 (480, 5040)	4970 (2220, 8775)	195 (120, 310)
Private	615	0 (0, 0)	840 (240, 1920)	1440 (0, 3360)	2940 (1080, 5920)	270 (120, 480)
Grade of study						
11	331	0 (0, 0)	1020 (80, 2160)	1680 (120, 4200)	3360 (1200, 7200)	240 (120, 390)
12	614	0 (0, 0)	1200 (276, 2175)	1680 (240, 3840)	3540 (1400, 6879)	240 (120, 380)
Subject of study						
Education	171	0 (0, 0)	1260 (160, 3080)	1920 (300, 5040)	4480 (1680, 9240)	210 (120, 320)
Humanities	24	0 (0, 0)	2010 (0, 3465)	1272 (0, 5760)	5216 (2430, 12,984)	205 (135, 358)
Management	298	0 (0, 0)	1112 (60, 2160)	1920 (110, 4920)	3790 (1272, 7840)	212 (120, 332)
Science	452	0 (0, 0)	1030 (280, 1960)	1520 (240, 3090)	3070 (1260, 5580)	288 (120, 480)
Time of study						
Morning	372	0 (0, 0)	1440 (120, 2952)	2340 (300, 5575)	5040 (1680, 8880)	210 (120, 315)
Day	573	0 (0, 0)	960 (240, 1870)	1440 (120, 3120)	2940 (1140, 5520)	270 (120, 480)
Environmental variables						
Mode of transport to school						
Walking	339	0 (0, 0)	1200 (240, 2240)	1680 (80, 4380)	3760 (1440, 7840)	255 (120, 390)
Cycle	134	0 (0, 0)	1960 (937, 3360)	2516 (1080, 4800)	5300 (3240, 9240)	225 (120, 338)
Motorcycle/Four-wheeled	472	0 (0, 0)	840 (50, 1680)	1440 (20, 3360)	2910 (960, 5600)	240 (120, 390)
Extracurricular activities at school						
Yes	654	0 (0, 0)	854 (240, 2100)	1648 (120, 3576)	3260 (1212, 6600)	236 (120, 375)
No	291	0 (0, 0)	1680 (280, 2520)	1840 (280, 4800)	3940 (1680, 7920)	260 (135, 401)
Playground at school						
Yes	793	0 (0, 0)	1040 (240, 2160)	1560 (80, 3480)	3300 (1200, 6700)	240 (120, 370)
No	152	0 (0, 0)	1560 (180, 2520)	3180 (840, 5490)	4740 (2540, 8400)	258 (120, 480)
Playground or park around home						
Yes	645	0 (0, 0)	1200 (240, 2240)	1720 (320, 4260)	3720 (1560, 7320)	252 (120, 390)
No	300	0 (0, 0)	1060 (240, 2100)	1340 (0, 3030)	3050 (1080, 6315)	205 (120, 363)
Adequate space to play or walk around home						
Yes	709	0 (0, 0)	1200 (240, 2240)	1720 (250, 4110)	3760 (1560, 7240)	240 (120, 390)
No	236	0 (0, 0)	900 (240, 1950)	1320 (0, 3330)	2910 (930, 6315)	198 (120, 365)
Family support to physical activity						
Yes	890	0 (0, 0)	1120 (240, 2175)	1680 (195, 3997)	3562 (1320, 7080)	240 (120, 390)
No	55	0 (0, 0)	720 (0, 2160)	1360 (0, 2660)	2880 (1120, 6320)	240 (120, 360)
Peer support to physical activity						
Yes	900	0 (0, 0)	1120 (240, 2240)	1680 (240, 4039)	3600 (1400, 7080)	240 (120, 380)
No	45	0 (0, 0)	720 (280, 1890)	1360 (0, 2650)	2160 (852, 5540)	240 (143, 440)
Lifestyle-related variables						
Current smoker						
Yes	30	0 (0, 0)	1680 (790, 3420)	3360 (1055, 6030)	5820 (3360, 9040)	238 (146, 394)
No	915	0 (0, 0)	1080 (240, 2160)	1680 (180, 3920)	3360 (1260, 6872)	240 (120, 385)
Current drinker						
Yes	42	0 (0, 0)	1560 (180, 2520)	3380 (1620, 5190)	5820 (3390, 8235)	240 (143, 391)
No	903	0 (0, 0)	1120 (240, 2160)	1680 (160, 3840)	3360 (1260, 6840)	240 (120, 380)
Screen time						
Moderate	599	0 (0, 0)	1120 (280, 2160)	1560 (240, 3360)	3340 (1260, 6480)	220 (120, 366)
Excessive	346	0 (0, 0)	1112 (30, 2520)	2190 (160, 5040)	4080 (1560, 7995)	270 (150, 405)

Note: Sitting time does not include the time spent during school hours

^a^Participants were first asked if they do any paid or unpaid work outside home. Work related physical activity is based only on the responses of participants who reported that they work outside home

### Domains of physical activity

Recreation domain contributed the most (47.09%) to the total physical activity score followed by travel domain (38.12%) and work domain (14.79%). Work-related physical activity was the least contributor because most of our participants were not engaged in any paid or unpaid works outside of their home. Participants had a travel-related median physical activity of 1120 MET-minutes/week and recreation-related median physical activity of 1680 MET-minutes/week (Table 3).

### Correlates of LPA among males

Among males, there was ethnic variation in physical activity engagement; respondents of minority ethnic groups were around 2.6 times more likely (OR: 2.65, 95% CI: 1.07, 6.56) to report LPA compared to Brahmin/Chhetri. Similarly, respondents who did not have playground or park around home were about 2.8 times more likely (OR: 2.82, 95% CI: 1.27, 6.28) to report LPA compared to those who had playground or park around home. Respondents who did not have family support were about 4.3 times more likely (OR: 4.27, 95% CI: 1.27, 14.30) to report LPA than those who had family support. Respondents who consumed alcohol were three times more likely (OR: 2.97, 95% CI: 1.03, 8.55) to report LPA compared to those who didn’t (Table 4).

**Table 4:** Odds ratio for low physical activity compared to moderate to vigorous physical activity stratified by sex

	Male (*n* = 471)			Female (*n* = 474)		
	n	Unadjusted OR (95% CI)	Adjusted OR (95% CI)^a^	n	Unadjusted OR (95% CI)	Adjusted OR (95% CI)^a^
Socio-demographic variables						
Age						
15–17 years	269	0.98 (0.12–7.98)	0.99 (0.12–8.62)	347	2.75 (0.61–12.49)	2.68 (0.56–12.87)
17–19 years	191	0.73 (0.09–6.15)	0.68 (0.08–6.05)	113	2.81 (0.60–13.20)	2.45 (0.50–11.95)
19–21 years	11	1	1	14	1	1
Ethnicity						
Brahmin/Chhetri	301	0.51 (0.23–1.12)	0.38 (0.15–0.94)	274	1.03 (0.53–2.02)	0.91 (0.44–1.86)
Aadibasi/Janajati	96	0.43 (0.15–1.23)	0.38 (0.13–1.14)	149	1.59 (0.79–3.20)	1.43 (0.70–2.94)
Others	74	1	1	51	1	1
Family type						
Nuclear	350	0.97 (0.45–2.05)	1.05 (0.48–2.28)	332	0.76 (0.50–1.15)	0.78 (0.51–1.20)
Non-nuclear	121	1	1	142	1	1
Educational status of father						
Illiterate	23	0.46 (0.06–3.60)	0.50 (0.05–4.86)	17	0.90 (0.31–2.67)	0.95 (0.27–3.43)
Primary	71	0.76 (0.27–2.15)	0.73 (0.21–2.52)	51	0.90 (0.46–1.76)	0.96 (0.41–2.26)
Secondary	201	0.87 (0.42–1.79)	0.85 (0.35–2.03)	197	0.97 (0.64–1.48)	0.85 (0.51–1.41)
High school and above	176	1	1	209	1	1
Educational status of mother						
Illiterate	85	0.79 (0.26–2.37)	0.71 (0.17–2.92)	56	0.98 (0.48–1.98)	1.01 (0.39–2.58)
Primary	107	0.84 (0.30–2.33)	0.84 (0.24–2.98)	77	1.11 (0.60–2.07)	1.08 (0.50–2.33)
Secondary	188	0.97 (0.40–2.35)	0.97 (0.34–2.72)	220	1.20 (0.74–1.94)	1.22 (0.70–2.13)
High school and above	91	1	1	121	1	1
Academic variables						
Type of school						
Public	160	0.41 (0.18–0.96)	0.49 (0.19–1.24)	170	0.59 (0.39–0.91)	0.47 (0.27–0.84)
Private	311	1	1	304	1	1
Grade of study						
11	168	0.93 (0.46–1.88)	1.90 (0.72–5.00)	163	1.57 (1.05–2.34)	0.98 (0.53–1.84)
12	303	1	1	311	1	1
Subject of study						
Education	65	0.13 (0.02–0.94)	0.05 (0.01–0.60)	106	1.15 (0.68–1.95)	1.66 (0.71–3.92)
Humanities	9	–	–	15	1.06 (0.33–3.48)	1.84 (0.45–7.57)
Management	153	0.56 (0.26–1.20)	0.15 (0.03–0.85)	145	2.06 (1.31–3.25)	2.20 (0.92–5.30)
Science	244	1	1	208	1	1
Time of study						
Morning	170	0.52 (0.24–1.14)	3.57 (0.66–19.18)	202	1.14 (0.77–1.69)	1.01 (0.48–2.13)
Day	301	1	1	272	1	1
Environmental variables						
Mode of transport to school						
Walking	174	0.62 (0.30–1.28)	0.70 (0.30–1.61)	165	0.98 (0.65–1.48)	0.98 (0.60–1.59)
Cycle	82	0.32 (0.09–1.09)	0.28 (0.06–1.26)	52	0.21 (0.08–0.54)	0.22 (0.08–0.62)
Motorcycle/Four-wheeled	215	1	1	257	1	1
Extracurricular activities at school						
Yes	293	0.82 (0.42–1.61)	1.02 (0.44–2.37)	361	1.12 (0.71–1.18)	0.99 (0.60–1.63)
No	178	1	1	113	1	1
Playground at school						
Yes	348	0.99 (0.47–2.10)	1.22 (0.47–3.16)	445	0.99 (0.44–2.25)	1.31 (0.51–3.37)
No	123	1	1	29	1	1
Playground or park around home						
Yes	325	0.42 (0.21–0.81)	0.36 (0.16–0.79)	320	0.70 (0.46–1.05)	0.59 (0.37–0.94)
No	146	1	1	154	1	1
Adequate space to play or walk around home						
Yes	353	0.62 (0.30–1.25)	1.08 (0.46–2.51)	356	0.84 (0.54–1.31)	1.15 (0.68–1.94)
No	118	1	1	118	1	1
Family support to physical activity						
Yes	446	0.25 (0.09–0.66)	0.23 (0.07–0.79)	444	0.89 (0.41–1.96)	0.73 (0.29–1.80)
No	25	1	1	30	1	1
Peer support to physical activity						
Yes	450	0.25 (0.09–0.74)	0.29 (0.08–1.06)	450	1.10 (0.45–2.70)	1.12 (0.39–3.19)
No	21	1	1	24	1	1
Lifestyle-related variables						
Current smoker						
Yes	29	1.34 (0.39–4.65)	1.96 (0.47–8.21)	1	–	–
No	442	1	1	473	–	–
Current drinker						
Yes	41	2.65 (1.09–6.46)	2.97 (1.03–8.55)	1	–	–
No	430	1	1	473	–	–
Screen time						
Moderate	294	1.04 (0.52–2.06)	0.88 (0.40–1.95)	305	0.70 (0.47–1.04)	0.69 (0.44–1.07)
Excessive	177	1	1	169	1	1

Odds ratio of smoking and alcohol consumption among females are blank because of the insufficient number of observations in a given cell

^a^Odds ratio for age, ethnicity, family type, educational status of father and educational status of mother were adjusted for socio-demographic variables. Odds ratio for type of school, grade of study, subject of study and time of study were adjusted for socio-demographic and academic factors. Odds ratio for mode of transport, extracurricular activities at school, playground at campus, playground or park around home, adequate space to play or walk around home, family support and peer support were adjusted for socio-demographic, academic and environmental factors. Socio-demographic, academic and environmental factors were adjusted for each of the life-style related factor

### Correlates of LPA among females

Among females, respondents of private school were around twice more likely (OR: 2.11, 95% CI: 1.20, 3.74) to report LPA than respondents of public school. Compared to bicycle commuters, those who used motorcycle or four-wheeled vehicle were 4.5 times more likely (OR: 4.54, 95% CI: 1.61, 12.5) to report LPA. Similarly, respondents who did not have playground or park around home were 1.7 times more likely (OR: 1.70, 95% CI: 1.06, 2.72) to report LPA compared to those who reported of having a playground or park around their homes (Table 4).

### Sedentary behavior

The mean sitting time was 282.93 min per day (SD = 206.90). Among males, it was 280.04 min per day (SD = 209.56) whereas it was 285.81 min per day (SD = 204.40) for females (more in Additional file 1).

## Discussion

We carried out a cross-sectional study to assess the level of physical activity, its correlates and the sedentary behavior of high school students in an urban district of Nepal. We found that one out of five respondents reported LPA. A large proportion of respondents met the criteria for WHO recommended physical activity level though the proportion among females was lower. We found gender disparity in physical activity. The respondents primarily engaged in physical activity for recreational purposes. Most of the correlates of low physical activity were different for adolescent males and females.

A review of the physical activity prevalence among Asian adolescents reported low levels of physical activity across countries [28]. However, they also cautioned that it is difficult to accurately estimate the prevalence given the absence of large number of studies and standardized and reliable measurement tools. While our study found that 31% of the adolescents did not meet the physical activity level recommended by WHO, recent studies on physical activity levels among adolescents in Bangladesh and India reported lower prevalence [29, 30]. This might be because of the methodological differences in the studies and the variation in socio-cultural environment. Similarly, the adult prevalence of LPA observed in a peri-urban setting in Nepal was 43% [19] -- much higher than what we observed among young adults in this study. Given that LPA contributes to 4.1% of all-cause mortality in Nepal [31], a high prevalence of LPA demands timely attention. Nepal implemented the Package of Essential Non-communicable Diseases Interventions (PEN) in 2016 which was developed by WHO for primary care setting. The package is currently being rolled out across the country [32]. Nepal NCDI Poverty Commission also recommends mass media campaigns for physical activity and healthy eating as one of the interventions at the local level for the control of non-communicable diseases [33].

The gender disparity in physical activity is a persistent finding in the global as well as national literature [19, 34, 35]. Though there was no significant gender difference in the sitting time, we found that females were five times more likely to report LPA. It is likely that females are engaged in physical activities not measured in the study such as housework. In many LMICs including Nepal, females are typically engaged more in unpaid household chores [36, 37]. As such, household chores which are important part of daily physical activities should be explored further in Nepalese context. This may also highlight the need to revise physical activity measure in LMICs to reflect gender differences in household work.

In the subgroup analysis, we observed that a majority of the determinants of LPA, except for the absence of a playground or park around their homes, varied by gender. This highlights the need for diverse interventions targeting males and females for the promotion of physical activity. Interestingly, in contrast to the previous study among school adolescents in Nepal which found no association between leisure time physical activity (LTPA) and school type [10], we found that students from private school are more likely to report LPA. The sitting time is also significantly higher for private school students than public school students. In Nepal, unlike private schools, public schools are characterized by teacher absenteeism, poor infrastructures, lower quality of education and unsatisfactory academic performance [38]. But private schools tend to spend more on the students’ academic achievement without much regard to the physical facilities and recreational activities. Moreover, public schools have shorter school hours, longer breaks and irregular classes which allow students time to engage in recreational activities [10, 38].

Similar to the findings of several studies [39, 40], our study also underlines the role of active transport such as cycling to school. However, students’ mode of transport depends on the distance between home and school. Nonetheless, development of an environment conducive to the routine physical activity is crucial. Evidence on physical activity research shows that measures like building running tracks and playgrounds, safe cycling and walking lanes, discouraging television viewing are effective approaches to promote physical activity [41]. Besides, interpersonal factors such as support from parents and family members has a significant influence on the physical activity of adolescents [42]. Therefore, it is important to aware families and communities about the benefits of routine physical activity.

Our study is the first to explore physical activity level in the district. This study can be useful to fill the information gap on the determinants of LPA in LMICs as well as to inform ongoing and forthcoming policies and interventions on promoting physical activity. However, we acknowledge that our study has a few limitations. First, we enrolled school-going adolescents only. Given the net enrolment rate of 14.4% in higher secondary level in the district [43], the findings might not be generalizable to overall adolescent population in the district. Moreover, our sampling strategy included schools with at least 35 students in each grade. Therefore, it fails to capture if there are systematic differences between smaller schools and larger schools in terms of PA levels and correlates. Second, our assessment of LPA may be an underestimation. GPAQ classification is based on the criteria set by WHO for people aged 18–64 years while our study population represents a mixed age group ranging from 15 to 21 years. Third, we observe that the 95% confidence interval for many effect measures were too wide to claim correlation with certainty, probably because our sample size, though large, was not sufficient enough for the huge number of potential correlates we explored in this study. Fourth, lack of any item on cellphone/tablet use for assessment of screen time may have influenced the actual amount of screen time. And lastly, our estimation of physical activity level relies on the information provided by the students about their routine activities. While every effort was made to assure that the students understand the questions and respond accurately, recall and social-desirability bias might have been present.

## Conclusions

Our findings indicate that one among seven adolescents in a south-western district of Nepal does not meet the WHO recommendations on physical activity for health. School type, grade of study, mode of transport, family support, and availability of playground/park around home were identified as the correlates of LPA. Most of the correlates were different for males and females. The nature of the research does not allow us to recommend definitive interventions, however, we suggest that a multitude of factors needs to be considered when designing interventions to promote physical activity in school. This study will inform health personnel, school administration and policy makers about the scenario surrounding physical activity among higher secondary student population and may help to generate awareness and encourage further research.

## Additional file

Additional file 1: SedentaryBehaviorAnalysis_and_Questionnaire-LocalVersion. (DOCX 1525 kb)

## Data Availability

The datasets used and/or analyzed during the current study are available from the corresponding author on reasonable request.

## Abbreviations

DEO: District Education Office
EM: Expectation Maximization
GPAQ: Global Physical Activity Questionnaire
LMIC: Low and Middle-Income Country
LPA: Low physical activity
MET: Metabolic Equivalent
MVPA: Moderate to vigorous physical activity
NCD: Non-communicable disease
NDHS: Nepal Demographic and Health Survey
NHRC: Nepal Health Research Council
PEN: Package of essential non-communicable diseases
SD: Standard deviation
SEE: Secondary Education Examination
TPA: Total Physical Activity
WHO: World Health Organization

## References

[1] Penedo FJ, Dahn JR (2005). Exercise and well-being: a review of mental and physical health benefits associated with physical activity. Curr Opin Psychiatry.

[2] Wankel LM, Berger BG (1990). The psychological and social benefits of sport and physical activity. J Leis Res.

[3] Löllgen H, Böckenhoff A, Knapp G (2009). Physical activity and all-cause mortality: an updated meta-analysis with different intensity categories. Int J Sports Med.

[4] Salvy SJ, Roemmich JN, Bowker JC, Romero ND, Stadler PJ, Epstein LH (2009). Effect of peers and friends on youth physical activity and motivation to be physically active. J Pediatr Psychol.

[5] Dumith SC, Gigante DP, Domingues MR, Kohl HW (2011). Physical activity change during adolescence: a systematic review and a pooled analysis. Int J Epidemiol.

[6] World Health Organization. Physical activity. https://www.who.int/news-room/fact-sheets/detail/physical-activity. Accessed 18 Dec 2018.

[7] Ministry of Health, New Era (Firm) (2017). Nepal demographic and health survey 2016.

[8] Aryal KK, Mehata S, Neupane S, Vaidya A, Dhimal M, Dhakal P (2015). The burden and determinants of non communicable diseases risk factors in Nepal: findings from a nationwide STEPS survey. PLoS One.

[9] World Health Organization. Global recommendations on physical activity for health. Geneva: World Health Organization; 2010. 26180873

[10] Paudel S, Subedi N, Bhandari R, Bastola R, Niroula R, Poudyal AK (2014). Estimation of leisure time physical activity and sedentary behaviour among school adolescents in Nepal. BMC Public Health.

[11] Bloemen MAT, Backx FJG, Takken T, Wittink H, Benner J, Mollema J (2015). Factors associated with physical activity in children and adolescents with a physical disability: a systematic review. Dev Med Child Neurol.

[12] Gordon-Larsen P, McMurray RG, Popkin BM (2000). Determinants of adolescent physical activity and inactivity patterns. Pediatrics.

[13] Lippo BR d S, Silva IM d, Aca CRP, Lira PIC d, Silva GAP d, Motta MEFA (2010). Determinants of physical inactivity among urban adolescents. J Pediatr.

[14] Uijtdewilligen L, Nauta J, Singh AS, Van Mechelen W, Twisk JWR, Van Der Horst K (2011). Determinants of physical activity and sedentary behaviour in young people: a review and quality synthesis of prospective studies. Br J Sports Med.

[15] Nagata JM, Ferguson BJ, Ross DA (2016). Research priorities for eight areas of adolescent health in low- and middle-income countries. J Adolesc Health.

[16] Suwal BR (2014). Internal migration in Nepal.

[17] Qualtrics (2018). Calculating sample size.

[18] Armstrong T, Bull F (2006). Development of the World Health Organization global physical activity questionnaire (GPAQ). J Public Health.

[19] Vaidya A, Krettek A (2014). Physical activity level and its sociodemographic correlates in a peri-urban Nepalese population: a cross-sectional study from the Jhaukhel-Duwakot health demographic surveillance site. Int J Behav Nutr Phys Act.

[20] Mumu SJ, Ali L, Barnett A, Merom D (2017). Validity of the global physical activity questionnaire (GPAQ) in Bangladesh. BMC Public Health.

[21] Au TB, Blizzard L, Schmidt M, Pham LH, Magnussen C, Dwyer T (2010). Reliability and validity of the global physical activity questionnaire in Vietnam. J Phys Act Health.

[22] Bull FC, Maslin TS, Armstrong T (2009). Global physical activity questionnaire (GPAQ): nine country reliability and validity study. J Phys Act Health.

[23] World Health Organization. Global physical activity questionnaire (GPAQ) analysis guide. 1–23. https://www.who.int/ncds/surveillance/steps/resources/GPAQ_Analysis_Guide.pdf. Accessed 18 May 2019.

[24] World Health Organization. Global physical activity questionnaire (GPAQ). 1–25. https://www.who.int/ncds/surveillance/steps/GPAQ%20Instrument%20and%20Analysis%20Guide%20v2.pdf. Accessed 18 May 2019.

[25] Central Bureau of Statistics. National population and housing census 2011. Kathmandu: Government of Nepal; 2012.

[26] Scholaro Pro. Education system in Nepal. https://www.scholaro.com/pro/countries/Nepal/Education-System. Accessed 18 Dec 2018.

[27] Soley-bori M (2013). Dealing with missing data: key assumptions and methods for applied analysis.

[28] Müller AM, Khoo S, Lambert R (2013). Review of physical activity prevalence of Asian school-age children and adolescents. Asia Pac J Public Health.

[29] Khan A, Burton NW, Trost SG (2017). Patterns and correlates of physical activity in adolescents in Dhaka city, Bangladesh. Public Health.

[30] Balaji S, Karthik R, Durga R, Harinie S, Ezhilvanan M (2018). Intensity of physical activity among school going adolescents in Chennai, South India. Int J Community Med Public Health.

[31] Lee IM, Shiroma EJ, Lobelo F, Puska P, Blair SN, Katzmarzyk PT (2012). Effect of physical inactivity on major non-communicable diseases worldwide: an analysis of burden of disease and life expectancy. Lancet.

[32] Primary Health Care Revitalization Division. PEN Protocol. http://phcrd.gov.np/index.php/PEN-Protocol. Accessed 18 Dec 2018.

[33] The Nepal NCDI Poverty Commission (2018). National Report - 2018.

[34] Van Der Horst K, Paw MJCA, Twisk JWR, Van Mechelen W (2007). A brief review on correlates of physical activity and sedentariness in youth. Med Sci Sports Exerc.

[35] Bauman AE, Reis RS, Sallis JF, Wells JC, Loos RJF, Martin BW (2012). Correlates of physical activity: why are some people physically active and others not?. Lancet.

[36] Central Bureau of Statistics. Population monograph of Nepal: volume III (economic demography). Kathmandu: Government of Nepal; 2014.

[37] United Nations Children’s Fund. Harnessing the power of data for girls: taking stock and looking ahead to 2030. New York: UNICEF; 2016.

[38] Thapa A (2013). Does private school competition improve public school performance? The case of Nepal. Int J Educ Dev.

[39] Østergaard L, Kolle E, Steene-Johannessen J, Anderssen SA, Andersen LB (2013). Cross sectional analysis of the association between mode of school transportation and physical fitness in children and adolescents. Int J Behav Nutr Phys Act.

[40] Smith L, Sahlqvist S, Ogilvie D, Jones A, Corder K, Griffin SJ (2012). Is a change in mode of travel to school associated with a change in overall physical activity levels in children? Longitudinal results from the SPEEDY study. Int J Behav Nutr Phys Act.

[41] Kahn EB, Ramsey LT, Brownson RC, Heath GW, Howze EH, Powell KE (2002). The effectiveness of interventions to increase physical activity: a systematic review. Am J Prev Med.

[42] Morrissey JL, Janz KF, Letuchy EM, Francis SL, Levy SM (2015). The effect of family and friend support on physical activity through adolescence: a longitudinal study. Int J Behav Nutr Phys Act.

[43] Government of Nepal. Education in figures 2017 (at a glance). Kathmandu: Ministry of Education, Science & Technology; 2017.

