# Supplementary material for: Physical activity and its correlates among higher secondary school students in an urban district of Nepal
Source: BMC Public Health. 2019 Jul 5;19:886. doi: 10.1186/s12889-019-7230-2 (PMC6612167; doi:10.1186/s12889-019-7230-2)

**Physical activity categorization**

**High:** If number of days of vigorous or moderate physical activity at (work + travel + recreational activities) >= 7 days AND total physical activity MET minutes per week is >= 3000

**Moderate:** If number of days of vigorous or moderate physical activity at (work + travel + recreational activities) >= 5 days AND total physical activity MET minutes per week is >= 600

**Low:** If the value does not reach the criteria for either high or moderate levels of physical activity

**Sensitivity analysis**

To see how change in merging pattern effect association between physical activity and other variables, we did sensitivity analysis by calculating the odds of vigorous physical activity (VPA) compared to low to moderate physical activity (LMPA), a single category developed by merging the low and moderate physical activity. Among males, type of school, subject of study, time of study, mode of transport to school, and peer support were found to be associated with vigorous physical activity. However, after adjustment, time of study (OR: 2.47, 95% CI: 1.01, 6.03 for ‘morning’), mode of transport (OR: 2.59, 95% CI: 1.17, 5.76 for ‘cycle’), and peer support (OR: 2.94, 95% CI: 1.06, 8.17 for ‘yes’) retained the association. Similarly among females, mode of transport (OR: 3.49, 95% CI: 1.65, 7.39 for ‘cycle’) and playground or park around home (OR: 1.91, 95% CI: 1.15, 3.15 for ‘yes’) were found to be associated with vigorous physical activity after adjustment though type of school, subject of study, time of study, ethnicity, educational status of father, mode of transport, playground or park around home, and adequate space to play or walk around home were associated with vigorous physical activity during crude analysis.

**Table S1 Sedentary behavior/Sitting time analysis**

We employed Independent sample t-test or ANOVA or Welch’s t-test, when appropriate, to determine the mean difference in sitting time among males and females across different socio-demographic, academic, environmental, and lifestyle-related variables. Similarly, post hoc comparisons, using either Tukey’s HSD test or Games-Howell post hoc procedure, as appropriate, were done to determine which category differed significantly from other.

**Table 1: Sitting time comparison stratified by sex**

|  | **N** | **Sitting time (minutes per day)** | | | | | |
| --- | --- | --- | --- | --- | --- | --- | --- |
|  |  | **Male (n=471)** | | | **Female (n=474)** | | |
|  |  | **Mean (SD)** | **t or F-statistic** | **p** | **Mean (SD)** | **t of F-statistic** | **p** |
| **Socio-demographic variables** | | | | | | | |
| **Age** |  |  |  |  |  |  |  |
| 15-17 years | 616 | 284.34 (210.72) | F=0.38 | 0.685 | 291.12 (208.36) | Welch’s F=3.92 | 0.028 |
| 17-19 years | 304 | 276.81 (206.68) |  |  | 279.33 (200.35) |  |  |
| 19-21 years | 25 | 231.09 (242.85) |  |  | 206.43 (104.23) |  |  |
| **Ethnicity** |  |  |  |  |  |  |  |
| Brahmin/Chhetri | 575 | 293.91 (218.94) | Welch’s F=4.00 | 0.020 | 296.02 (206.54) | F=0.85 | 0.429 |
| Aadibasi/Janajati | 245 | 233.01 (173.45) |  |  | 269.47 (199.56) |  |  |
| Others | 125 | 284.62 (207.04) |  |  | 278.69 (207.39) |  |  |
| **Family type** |  |  |  |  |  |  |  |
| Nuclear | 682 | 273.29 (200.85) | t=1.19 | 0.235 | 280.81 (204.98) | t=0.81 | 0.416 |
| Non-nuclear | 263 | 299.56 (232.71) |  |  | 297.49 (203.27) |  |  |
| **Educational status of father** |  |  |  |  |  |  |  |
| Illiterate | 40 | 262.93 (187.19) | Welch’s F=2.92 | 0.038 | 162.88 (99.04) | Welch’s F=11.31 | <0.001 |
| Primary | 122 | 233.82 (172.37) |  |  | 228.55 (142.68) |  |  |
| Secondary | 398 | 269.72 (198.58) |  |  | 273.66 (189.55) |  |  |
| High school and above | 385 | 312.70 (233.42) |  |  | 321.24 (228.17) |  |  |
| **Educational status of mother** |  |  |  |  |  |  |  |
| Illiterate | 141 | 259.52 (194.51) | F=2.70 | 0.045 | 218.04 (138.87) | Welch’s F=4.98 | 0.002 |
| Primary | 184 | 244.28 (194.37) |  |  | 274.42 (192.92) |  |  |
| Secondary | 408 | 289.11 (209.59) |  |  | 288.79 (207.71) |  |  |
| High school and above | 212 | 322.50 (233.09) |  |  | 319.01 (224.00) |  |  |
| **Academic variables** | | | | | | | |
| **Type of school** |  |  |  |  |  |  |  |
| Public | 330 | 222.23 (150.81) | t=4.97 | <0.001 | 231.17 (143.75) | t=5.00 | <0.001 |
| Private | 615 | 309.78 (228.69) |  |  | 316.37 (226.01) |  |  |
| **Grade of study** |  |  |  |  |  |  |  |
| 11 | 331 | 288.33 (217.32) | t=0.64 | 0.523 | 313.38 (222.05) | t=2.13 | 0.033 |
| 12 | 614 | 275.44 (205.34) |  |  | 271.36 (193.32) |  |  |
| **Subject of study** |  |  |  |  |  |  |  |
| Education | 171 | 219.69 (138.87) | Welch’s F=8.61 | <0.001 | 260.44 (190.16) | Welch’s F=2.67 | 0.054 |
| Humanities | 24 | 221.11 (89.23) |  |  | 250.10 (131.82) |  |  |
| Management | 298 | 238.93 (162.38) |  |  | 264.84 (184.34) |  |  |
| Science | 452 | 324.07 (242.88) |  |  | 315.93 (225.09) |  |  |
| **Time of study** |  |  |  |  |  |  |  |
| Morning | 372 | 226.37 (134.34) | t=4.91 | <0.001 | 247.02 (161.69) | t=3.78 | <0.001 |
| Day | 573 | 310.35 (236.81) |  |  | 314.61 (227.10) |  |  |
| **Environmental variables** | | | | | | | |
| **Mode of transport to school** |  |  |  |  |  |  |  |
| Walking | 339 | 286.61 (197.69) | F=0.45 | 0.641 | 293.29 (218.99) | Welch’s F=2.40 | 0.095 |
| Cycle | 134 | 260.68 (202.67) |  |  | 238.67 (157.94) |  |  |
| Motorcycle/Four-wheeled | 472 | 282.11 (221.63) |  |  | 290.55 (202.46) |  |  |
| **Extracurricular activities at school** |  |  |  |  |  |  |  |
| Yes | 654 | 273.73 (208.18) | t=0.84 | 0.403 | 274.74 (204.80) | t=2.12 | 0.035 |
| No | 291 | 290.42 (211.97) |  |  | 321.18 (199.90) |  |  |
| **Playground at school** |  |  |  |  |  |  |  |
| Yes | 793 | 275.31 (203.52) | t=0.82 | 0.410 | 281.14 (198.19) | t=1.45 | 0.156 |
| No | 152 | 293.43 (226.14) |  |  | 357.42 (277.89) |  |  |
| **Playground or park around home** |  |  |  |  |  |  |  |
| Yes | 645 | 275.79 (197.94) | t=0.62 | 0.539 | 298.68 (201.49) | t=1.98 | 0.048 |
| No | 300 | 289.49 (233.80) |  |  | 259.06 (208.44) |  |  |
| **Adequate space to play or walk around home** |  |  |  |  |  |  |  |
| Yes | 709 | 286.41 (204.66) | t=1.09 | 0.276 | 283.15 (196.07) | t=0.49 | 0.624 |
| No | 236 | 260.97 (223.39) |  |  | 293.82 (228.40) |  |  |
| **Family support to physical activity** |  |  |  |  |  |  |  |
| Yes | 890 | 281.15 (210.43) | t=0.49 | 0.627 | 287.30 (206.39) | t=0.61 | 0.542 |
| No | 55 | 260.20 (196.24) |  |  | 263.73 (173.69) |  |  |
| **Peer support to physical activity** |  |  |  |  |  |  |  |
| Yes | 900 | 278.99 (207.30) | t=0.50 | 0.615 | 284.53 (205.89) | t=0.59 | 0.554 |
| No | 45 | 302.57 (258.14) |  |  | 309.88 (176.02) |  |  |
| **Lifestyle-related variables** | | | | | | | |
| **Current smoker** |  |  |  |  |  |  |  |
| Yes | 30 | 263.84 (158.11) | t=0.43 | 0.668 | 235.71* | t=0.25 | 0.806 |
| No | 915 | 281.10 (212.59) |  |  | 285.92 (204.60) |  |  |
| **Current drinker** |  |  |  |  |  |  |  |
| Yes | 42 | 291.79 (214.44) | t=0.38 | 0.708 | 300.00* | t=0.07 | 0.945 |
| No | 903 | 278.92 (209.31) |  |  | 285.78 (204.61) |  |  |
| **Screen time** |  |  |  |  |  |  |  |
| Moderate | 599 | 267.43 (216.21) | t=1.69 | 0.093 | 267.82 (200.80) | t=2.59 | 0.010 |
| Excessive | 346 | 300.98 (196.82) |  |  | 318.28 (207.40) |  |  |

*Single observation

**
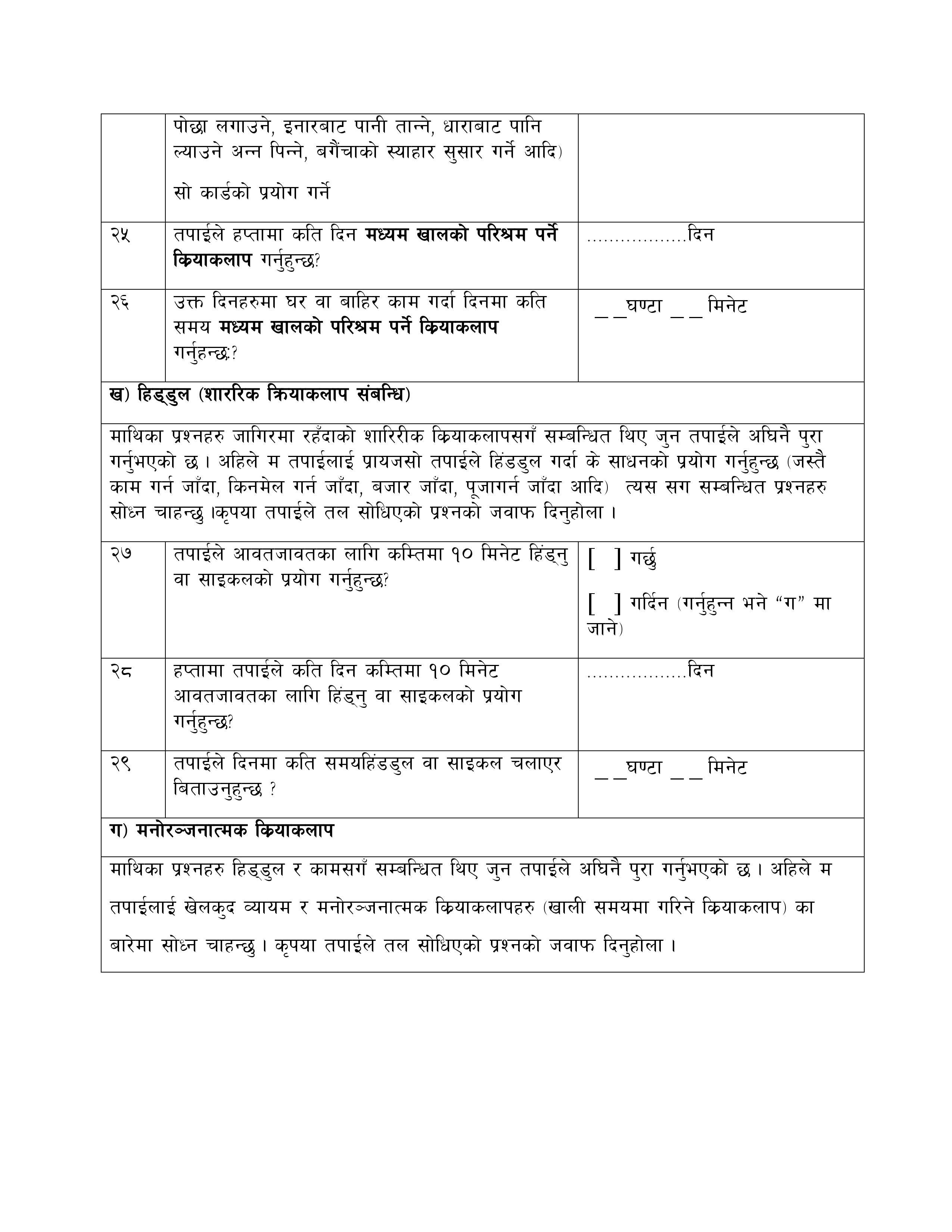
**


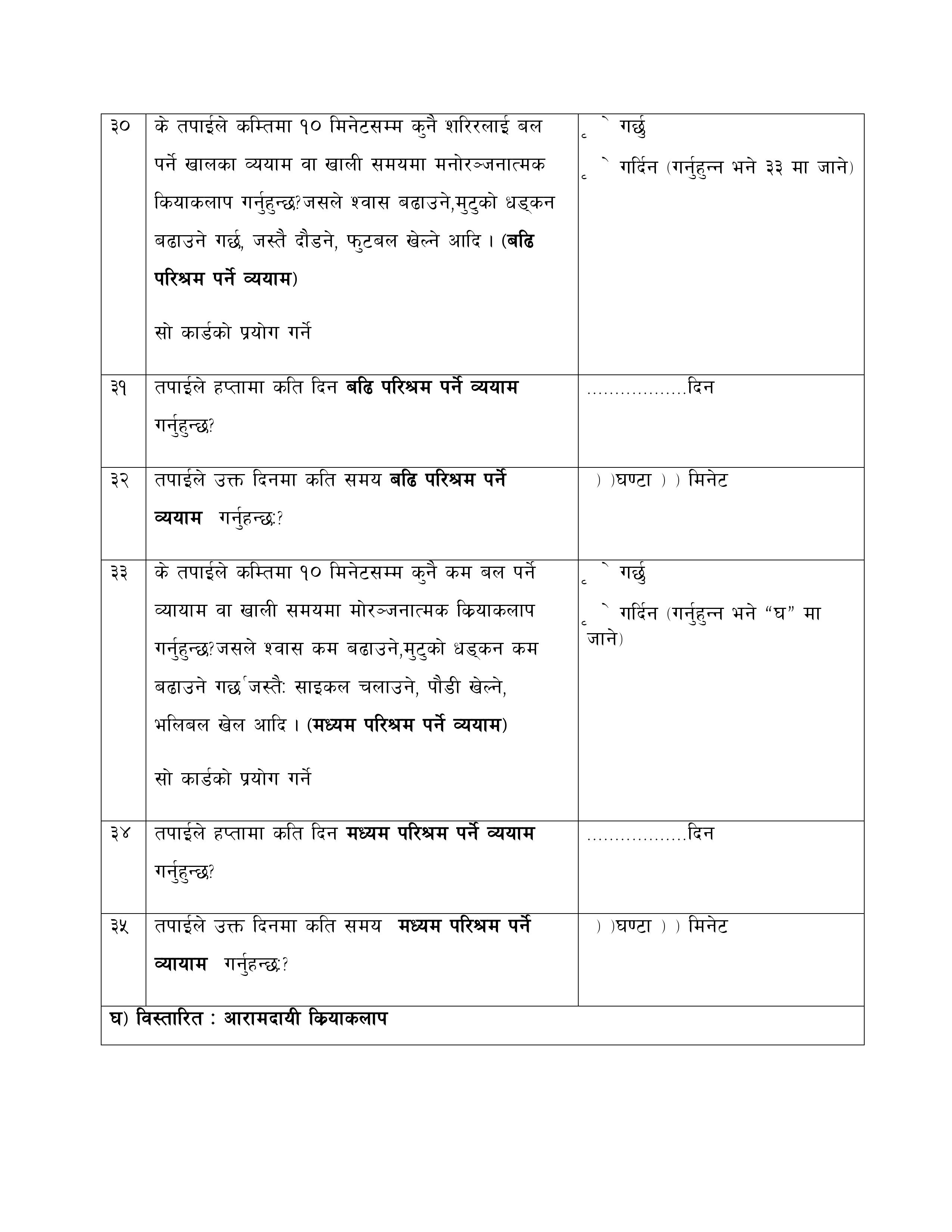


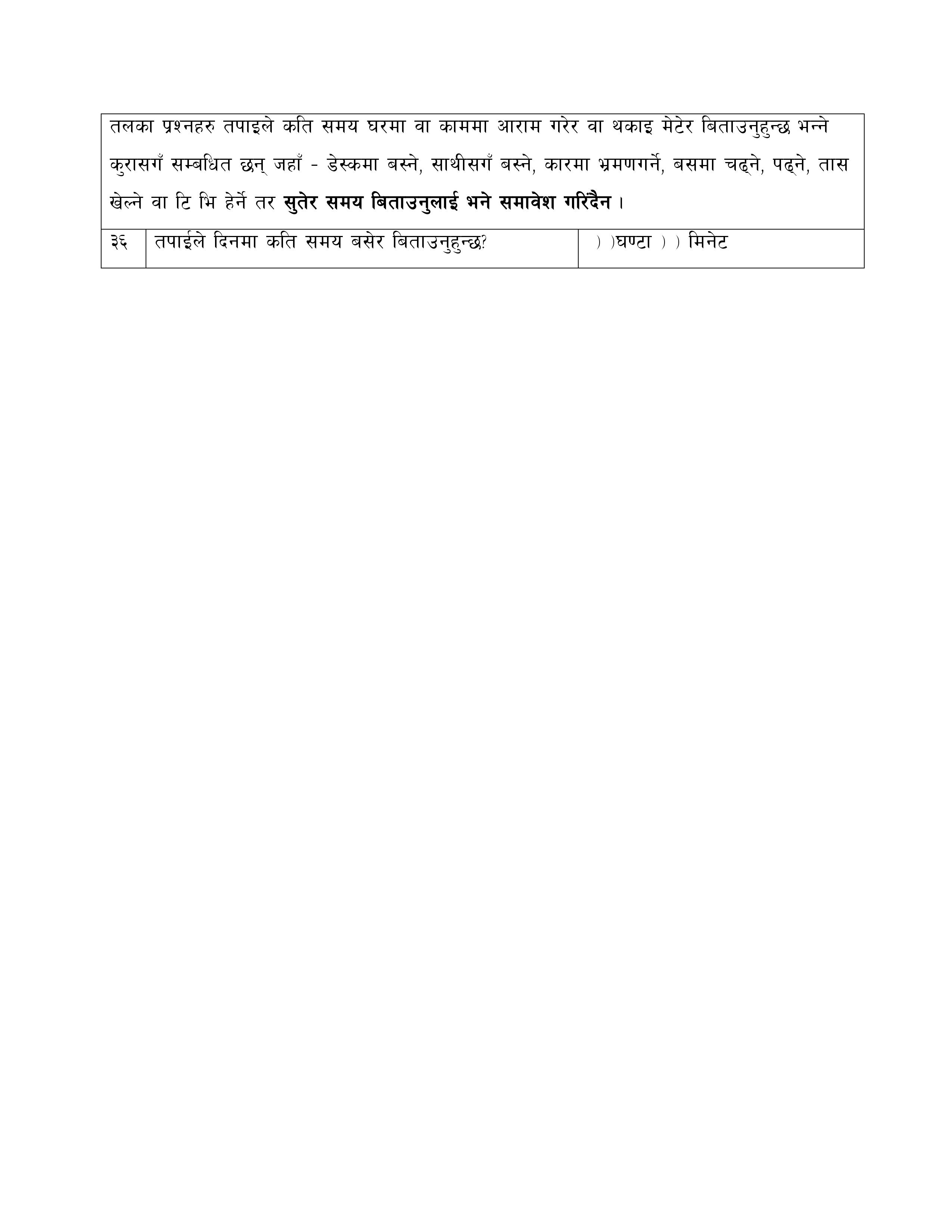

Supplement: Supplementary file 1 — SedentaryBehaviorAnalysis_and_Questionnaire-LocalVersion. (DOCX 1525 kb) [file 12889_2019_7230_MOESM1_ESM.docx]
